# Neuromuscular and perceptual-cognitive response to 4v4 small-sided game in youth soccer players

**DOI:** 10.3389/fphys.2023.1260096

**Published:** 2023-11-09

**Authors:** Filip Skala, Erika Zemková

**Affiliations:** ^1^ Department of Biological and Medical Sciences, Faculty of Physical Education and Sport, Comenius University in Bratislava, Bratislava, Slovakia; ^2^ Faculty of Health Sciences, University of St. Cyril and Methodius in Trnava, Trnava, Slovakia

**Keywords:** fatigue, agility, cognitive functions, reaction time, load management, soccer training

## Abstract

The physical and psychological load of small-sided games (SSGs) can affect players’ neuromuscular and cognitive functions. Yet, little is known about the acute performance changes after such a specific exercise in young soccer players and their association with exercise load applied. This study investigates i) the neuromuscular and perceptual-cognitive response to the SSG exercise load, and ii) the relationship between pre- and post-SSG changes in variables of performance and the respective exercise load in youth soccer players. Sixteen participants (13.6 ± 0.5 years) underwent a 30-min SSG 4v4 + GK protocol. Prior to and after the SSG they performed countermovement jump (CMJ), planned and reactive Y-shaped agility tests (PA, RA), and go/no-go task (GNG). Their subjective perception of fatigue was evaluated by visual analog scale. Fatigue induced by SSG (perception of fatigue increased by 41.56%, *p* = .001, g = 4.15) increased PA time (4.04%, *p* = .002, g = .97), RA time (6.45%, *p* = .003, g = 1.16), and number of errors in the response inhibition task (87.1%, *p* = .023, r_c_ = .57), whilst decreased CMJ height (−6.65%, *p* = .014, g = .56). These performance deteriorations were not significantly associated with neither internal nor external load variables. However, a less pronounced drop in performance was related to external load variables, i.e., ∆CMJ height and ∆RA time correlated with very high-speed running (r_s_ = .66, *p* = .006; r_s_ = −.50, *p* = .022; respectively) and maximal speed (*r* = .54, *p* = .032; *r* = −.52, *p* = .037; respectively), whilst ∆PA time was associated with high-intensity accelerations (r_s_ = −.76, *p* = .002). These findings indicate that fatigue induced by SSG affects both planned and reactive agility, decision-making in response inhibition task, and explosive strength in youth soccer players regardless of significant contribution of any robust internal or external load variables. Nonetheless, high-intensity actions within SSG partially compensate for the decrements in their agility performance and explosive strength. The load variables encountered during SSG do not fully reflect youth players’ neuromuscular and perceptual-cognitive responses to sport-specific exercise.

## 1 Introduction

Small-sided games (SSGs) provide an effective tool for conditioning team sport players in a specific game environment (Gabbett, 2006). Their intermittent character produces a relatively high neuromuscular and metabolic load (Owen et al., 2012; Sarmento et al., 2018), which can induce temporal decrements in players’ physical performance (Faude et al., 2014; Martínez-Serrano et al., 2023). SSGs also demand players’ cognitive functions (Figueira et al., 2019; Mitrotasios et al., 2021). Therefore, proper prescription of SSGs and respective exercise load monitoring is important for the performance optimization of soccer players (Branquinho et al., 2021a; Branquinho et al., 2021b).

Crucial abilities for soccer performance, such as explosive strength, sprinting, and change of direction speed (Rampinini et al., 2007; Nygaard Falch et al., 2019), all depend on the neuromuscular system. Neuromuscular performance temporarily decreases in parallel with accumulated exercise load in SSGs (Rebelo et al., 2016; Bujalance-Moreno et al., 2020). This is mainly contributed to the high amount of mechanical work produced by numerous in-game changes in speed and direction of movement, in terms of high-intensity accelerations and decelerations (Gaudino et al., 2014). External load is to a high extent responsible for muscle damage (Silva et al., 2018). The impairment of muscle cells’ contractile functions results in lower muscle force production (Thorlund et al., 2009). This neuromuscular fatigue can also be accentuated by acute depletion of muscles’ energy stores (i.e., glycogen, phosphocreatine) as a result of intermittent exercise (Gaitanos et al., 1993; Reilly et al., 2008). Temporal performance declines induced by sport-specific exercise load are typical for players’ explosive strength and speed (Katis and Kellis, 2009; Rebelo et al., 2016; Rowell et al., 2017).

Besides the high neuromuscular and metabolic demands of SSGs, sufficient attention, perception, and visual information processing from a dynamic environment are of special importance for soccer players (Klatt and Smeeton, 2022). Cognition needs to be activated more often and in different ways during situations experienced in the game (Rodrigues et al., 2022). Cognitive performance can be enhanced by acute bouts of exercise (Dupuy et al., 2018). Positive effects may be attributed to the sympathetic functions increasing heart rate, levels of excitatory neurotransmitters in the brain, and cortisol secretion (Tomporowski, 2003). On the contrary, physical and mental effort can also produce negative effects on players’ perceptual and cognitive performance (Skala and Zemková, 2022). These effects are often ascribed to the increased levels of brain catecholamines (McMorris et al., 2008) and limited activation in centers responsible for higher-order cognitive functions (Dietrich, 2006).

Several studies reported a decline in cognitive performance following acute bouts of exercise (Del Giorno et al., 2010; Donnan et al., 2022; Teoldo et al., 2022). For example, longer reaction time and impaired object detection were found in sport-specific visual tasks (Frýbort et al., 2016; Klatt and Smeeton, 2021). However, these declines may be task-specific and related to the time of post-exercise testing (Moore et al., 2012). Accumulated repetitions of SSGs were found to impair decision-making ability in terms of progressive deterioration of passing accuracy (Mitrotasios et al., 2021). In addition, decision-making and perception are important factors of agility performance in invasive sports (Young et al., 2015). The decline in agility performance occurs sooner when players react to external cues than when changing the direction of movement without reactions to visual stimuli (Ciocca et al., 2022). However, discrepancies in the literature exist regarding the effects of exercise on reactive and planned agility (Almonroeder et al., 2020).

Nevertheless, recent literature reviews have shown that the majority of studies explore the acute effects of exercise on performance of adult and adolescent athletes (Skala and Zemková, 2022; Teoldo et al., 2022). Children are more resistant to neuromuscular fatigue and recover faster from high-intensity physical exertion compared to adults (Falk and Dotan, 2006; Ratel et al., 2006). Yet, little is known about the effects of SSGs in youth players and their neuromuscular and perceptual-cognitive performance under fatigue conditions. Since SSGs are widely used in both adult and youth soccer training, it is of our interest to investigate i) the neuromuscular and perceptual-cognitive performance response to the SSG exercise load, and ii) the relationship between pre- and post-SSG changes in variables of performance and respective exercise load in youth soccer players. Here, we hypothesized neuromuscular and perceptual-cognitive performance impairment after the SSG. While the external load variables would be associated with decrements in explosive strength and agility, the internal load variables would be related to impaired accuracy during the response inhibition task.

## 2 Materials and methods

### 2.1 Participants

Sixteen youth soccer players (13.6 ± 0.5 years; 163.4 ± 5.9 cm; 50.4 ± 7.1 kg; Table 1) from a local first-tier academy were voluntary recruited to participate in this study. All participants had a minimum of 5-year experience with the academy soccer training. They performed 4 training sessions (80–100-min) per week and played an official eleven-a-side match (2 × 35-min) during the weekend on the regular-sized soccer field. Two goalkeepers participated in the SSG, but were not included in the analysis. The players were asked to avoid physical activity for 24-h prior to the experiment and to sleep at least 8-h a night. They were instructed to consume water and light meal 2–3-h before the testing session. Participants were free from neuromuscular injuries or any disorders. Written informed parental consent was obtained before the study. Each participant and his parents agreed to participate in the experiment and were notified about the withdrawal from the study at any time. The procedures followed were in accordance with the ethical standards on human experimentation stated in compliance with the 1964 Helsinki Declaration and its later amendments. The project was approved by the ethics committee of the Faculty of Physical Education and Sport, Comenius University in Bratislava (no. 3/2022, date: 22 September 2022).

**TABLE 1:** Descriptive statistics for somatometric and physical fitness assessments of participants. SD (±), standard deviation; CV (%), coefficient of variation.

	Mean	SD (±)	CV (%)
Age (years)	13.6	0.5	3.7
Height (cm)	163.4	5.9	3.7
Body mass (kg)	50.4	7.1	15.5
10-m sprint (s)	1.88	0.06	3.0
Maximal speed (km/h)	26.5	1.8	6.6
Countermovement jump (cm)	30.5	3.7	12.1
Yo-Yo intermittent recovery 1 test (m)	1,299	216	16.6

### 2.2 Study design

This study was constituted as a quasi-experimental investigation without the random assignment of participants to conditions or their order. The pretest-posttest design was used. The dependent variable was measured once before the exercise was implemented and once after it was implemented. Measurements were performed as part of regular in-season training. One week before the experiment, the participants completed an assessment of body height, body mass, acceleration speed in a 10-m sprint, maximal speed obtained from the lowest 10-m split time in a 40-m run, explosive strength in a countermovement jump, and aerobic endurance in a Yo-Yo intermittent recovery test level 1 (Table 1).

**FIGURE 1:**
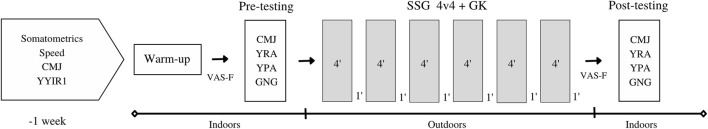
Experimental design. CMJ, countermovement jump; YYIR1, Yo-Yo intermittent recovery level 1; VAS-F, Visual analog scale of fatigue; YRA, Y-shaped reactive agility test; YPA, Y-shaped planned agility test; GNG, Go/No-go test; SSG 4v4 + GK, Small-sided game 4 vs. 4 + Goalkeepers.

Prior to the experiment, the participants underwent a warm-up that included low-intensity running, dynamic stretching, and running drills. No specific exercise with a ball was included. The warm-up and testing of neuromuscular and perceptual-cognitive performance were executed indoors to secure standardized conditions. The participants wore standard indoor soccer footwear. Afterwards they were individually asked to evaluate their subjective perception of fatigue. They changed the footwear to standard football boots and moved to the soccer field located right outside the testing room. The SSGs were performed outdoors on a regular artificial grass soccer field, between 3 and 6 p.m. in temperatures 7 to 10°C (Meteoblue, Basel, Switzerland). After the SSG protocol, the participants immediately moved to the testing room and underwent the same testing procedure within 10-min of its completion. Tests were executed in randomized order. A whole experimental design is shown in Figure 1.

### 2.3 Small-sided game

The SSG (4v4 + Goalkeepers) was performed on the outdoor soccer field with artificial grass using formal 11-a-side goals. The size of the pitch 40 × 25-m (length × width) provided a relative field space of 125 m^2^ per player (Calderón Pellegrino et al., 2020). The exercise included six 4-min bouts separated by 1-min passive recoveries (overall game time of 24-min). The players were divided into two teams by the head coach in accordance with his perception of their physical, technical, and tactical skills (Torrents et al., 2016). Standard 11-a-side soccer rules were followed except for the offside rule. Field players were able to pass to goalkeepers. Verbal encouragement from coaches was permitted, but not feedback related to the players’ technical and tactical performance. Several balls were located near the sidelines around the pitch to increase the effective play time (Hill-Haas et al., 2008). The players were familiar with this SSG format as it was used regularly in training.

### 2.4 Data collection

#### 2.4.1 Load monitoring

The physical activity profiles of players in SSG were assessed using 10-Hz GPS units with heart rate (HR) sensors Polar Team Pro (Polar, Kempele, Finland). This system is valid and reliable for the assessment of the external and internal load of soccer players (Scott et al., 2016; Akyildiz et al., 2022). Units were mounted with adjustable straps on the front of participants’ chests. The variables of the external load included total distance covered (TDC), number of accelerations (ACC), number of decelerations (DEC), maximal speed (MSP), and distance covered in speed zones. These zones were categorized as follows: low-speed running (LSR = 0.70–6.99 km h^−1^), medium-speed running (MSR = 7–13.99 km h^−1^), high-speed running (HSR = 14–20.99 km h^−1^), and very high-speed running (VHSR; >21 km h^−1^). Accelerations and decelerations were characterized as low to moderate intensity (LMACC and LMDEC; 1–2.99 m s^−2^), and high-intensity (HACC and HDEC; >3 m s^−2^). Internal load variables included absolute heart rate (HR_avg_) and relative heart rate (%HR_avg_). Relative HR zones were categorized as <59%, 60%–69%, 70%–79%, 80%–89%, and >90% HR_max_ (%). Relative HR percentages were calculated using the equation 208 – 0.7 × age (Tanaka et al., 2001; Nikolaidis, 2014).

#### 2.4.2 Subjective perception of fatigue

Participants were individually asked to evaluate their subjective perception of fatigue prior to and after the SSG by marking a vertical line on the 100-mm visual analog scale of fatigue (VAS-F). This type of assessment was previously found valid and reliable for assessing the subjective perception of fatigue (Lee et al., 1991). The scale was anchored with the words “no fatigue” on the left end, and “extremely fatigued” on the right end. The VAS-F score in an arbitrary unit (A.U.) was determined as the distance in millimeters from the left end to the marked vertical line (Abbasi et al., 2018).

#### 2.4.3 Countermovement jump

The countermovement jump (CMJ) height was assessed using a portable optical system OptoGait (Microgate, Bolzano, Italy). Participants were instructed to lower their body to a squat position with a knee joint at approximately 90° and jump as high as possible without stopping in a lower position while keeping their hands on their waist (Bosco et al., 1983). They were also asked to avoid lateral and frontal movements and to keep their legs straight during the flight and landing phases. Since CMJ was precisely described in the familiarization session, the highest values of two jumps prior to and after the SSG were analyzed. Jumps were separated by 30-s rest periods. Sufficient reliability was reported when assessing two repetitions of jump height using the same device as in our study (ICC = 0.97; CV = 5.1%) (Krzysztofik et al., 2021).

#### 2.4.4 Y-shaped agility tests

A single light-based timing system Witty GATE and LED indicator Witty SEM (Microgate, Bolzano, Italy) were used to evaluate participants’ reactive agility (RA) time. This test is valid and reliable for the assessment of agility in team sports players (Oliver and Meyers, 2009; Horníková et al., 2021). All gates were set up in 1.2-m height and width of 1.5-m. Participants started approximately 0.3-m behind the starting gate. They were instructed to sprint straight and change the direction of running through the gate made of cones which was 5-m apart from the starting gate. A LED indicator in front of the subject (i.e., 3-m apart from the cone gate) randomly displayed a green arrow to the left or right after 500-ms since passing the starting gate. Participants responded to this signal by running to the arrow-pointing gate. The final gates were located 5-m apart from the middle gate at 45° angles. In case of execution errors (e.g., fall, slide, run to the incorrect gate), they were allowed to repeat the trial. Participants performed three pre-SSG trials with 30 s of rest between the repetitions and two post-SSG trials. The fastest trials prior to and after the SSG were analyzed.

The planned agility (PA) time was evaluated by an identical setup as the Y-shaped reactive agility test, excluding the visual signal. Participants were instructed to sprint to the particular gate before the beginning of this test. Two trials for each side with a 30-s rest were performed before the SSG. Post-SSG trials were performed twice. The sides of post-SSG trials were selected based on the stimuli generated in the previously performed reactive agility test.

#### 2.4.5 Go/no-go task

Perceptual cognitive performance was evaluated using a customized computer-based response inhibition task. The Go/no-go task (GNG) was performed through the online software Psytoolkit v3.4.1 (Stoet, 2010; 2017). Testing consisted of one set of 20 familiarization trials, followed by one set before the SSG, and one set after the SSG. Each set included 50 randomly generated trials with a correct:error response ratio of 4:1. Fixation point was presented for 1000-ms in the center of a 13-inch computer screen. Presentations of go/no-go signals were separated by a 1000-ms interstimulus pause. Signals were shown as a green/red silhouette of a soccer player with a white “GO”/“NO-GO” symbol written in the middle of the image. Participants were instructed to press a spacebar button as soon as the green “go” signal appeared and to suppress the “no-go” signal. In case of an error, a pause of 2000-ms was included before the next fixation. The mean “go” trials response time in milliseconds (GNGt) and the number of trials with an error of commission (GNGe) were recorded as outcome measures. Trials with a response time of less than 150-ms were excluded from the analysis (Zhao et al., 2015).

### 2.5 Statistical analysis

Data are presented as mean ± SD. The coefficient of variation (CV%) and 90% confidence intervals (CI 90%) were calculated to describe the physiological response of players to the SSG and the kinematic profile in the SSG. The normality of data distribution was determined using the Shapiro-Wilk test. In the case of normal data distribution, the paired-sample *t*-test was performed. Otherwise, the Wilcoxon signed rank test was performed. Significance was set at *p* < 0.05 (2-tailed). The smallest worthwhile change was calculated as between subjects’ SD multiplied by 0.2 (Tomczak and Tomczak, 2014). The *post hoc* statistical power of the sample size was calculated with G*Power (Version 3.1.9.6, Institut für Experimentelle Psychologie, Düsseldorf, Germany). The power for the number of subjects within the study sample was 1 − β = 0.604 with α = 0.05. To compensate for the lower sample size power, additional methods were used. The effect sizes for Hedges’ g were as follows: <0.2 trivial; 0.2–0.5 small; 0.5–0.8 moderate; >0.8 large effect. The Cohens’ r (r_c_) for non-parametrical data was as follows: <0.1 small; 0.3–0.5 moderate; >0.5 large effect size. Uncertainties in the true effects of the respective conditions were evaluated by magnitude-based inferences using customized spreadsheets. Magnitudes of clear effect were considered according to the following scale: 25%–75%, possibly; 75%–95% likely; 95%–99%, very likely; >99% most likely (Hopkins, 2007). Differences in the pre- and post-SSG performance variables were expressed as the mean of individual differences (Δ = post-SSG–pre-SSG). Associations between individual performance changes (Δ) and exercise load variables were reported using the Pearson/Spearman correlation coefficient (r/r_s_) with lower and upper confidence intervals (90%). The correlation strength was interpreted as follows: r/r_s_ = <0.1, trivial; 0.1–0.3, small; 0.31–0.49, moderate; 0.5–0.69, large; 0.7–0.89, very large; and 0.9–1, perfect correlation. Additionally, the amount of explained variance (R^2^) was determined for the parametric data (Hopkins et al., 2009). Statistical analyses were performed using Graph Pad Prism software 9.5.0 (Graph Pad Software, San Diego, CA, United States).

## 3 Results

**FIGURE 2:**
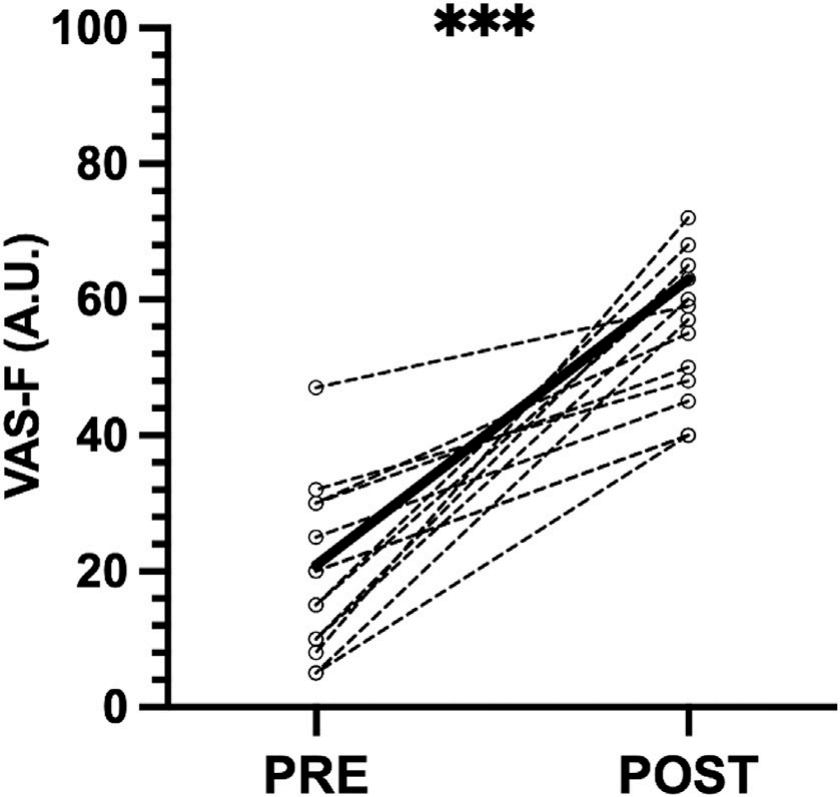
Individual pre- and post-SSG differences in perceived fatigue. Circles connected with dashed line indicate the individual data of each participant; black bold line indicates mean values. Significant changes between pre- and post-SSG assessment. ***p < 0.001. VAS-F, visual analog scale of fatigue.

The subjective perception of fatigue (VAS-F) increased after the SSG (41.56 ± 14.02 A.U, *p* = .001, g = 4.15, large effect; Figure 2). The internal and external load variables measured during SSG are shown in Table 2.

**TABLE 2:** Physiological response and kinematic profiles of players in the SSG protocol.

	MEAN	SD (±)	CI (90%)	CV (%)
HR_avg_ (bpm^−1^)	171.71	7.15	169, 175.5	4.2
HR_avg_ (%)	86.57	3.64	85, 88.3	4.2
TDC (m)	2,753	175.29	2,674, 2,834	6.4
TDC (m/min)	91.79	5.92	89.1, 94.5	6.4
<59% HR_max_ (%)	9.10	8.77	4.66, 13.7	96.4
60%–69% HR_max_ (%)	3.83	3.32	2.09, 5.57	87.7
70%–79% HR_max_ (%)	12.34	3.24	10.64, 14.04	26.3
80%–89% HR_max_ (%)	29.00	11.35	23.04, 34.97	43.43
>90% HR_max_ (%)	45.85	17.99	36.4, 55.29	43.52
LSR (m)	1,101	102.78	1,055, 1,148	9.3
MSR (m)	1,250	159.53	1,178, 1,322	12.8
HSR (m)	379	74.30	346.4, 425.9	19.6
VHSR (m)	13.50	11.57	8.3, 18.8	85.7
LMACC (n)	171.88	14.91	164.5, 180.7	8.7
HACC (n)	5.43	2.33	4.4, 6.4	42.9
LMDEC (n)	172.63	18.47	165.4, 178.5	10.7
HDEC (n)	9.75	4.89	7.9, 12.1	50.2
MSP (km/h)	22.32	1.53	21.49, 23.12	6.9

CI (90%), 90% lower and upper confidence intervals; CV%, coefficient of variation; HR_avg_ (bpm^−1^), average heart rate; HR_avg_ (%), average heart rate from maximum; HR_max_ (%), time spent in respective heart rate zone; TDC (m), total distance covered; TDC (m/min), relative total distance covered; LSR, low-speed running (0–6.99 km/h); MSR, medium-speed running (7–13.99 km/h); HSR, high-speed running (14–20.99 km/h); VHSR, very high-speed running (>21 km/h); LMACC, low to moderate accelerations (1–2.99 m/s); LMDEC, low to moderate decelerations (1–2.99 m/s); HACC, high-intensity accelerations (>3 m/s); HDEC, high-intensity decelerations (>3 m/s); MSP, maximal speed achieved during SSG.

### 3.1 Neuromuscular and perceptual-cognitive response to SSG

**FIGURE 3:**
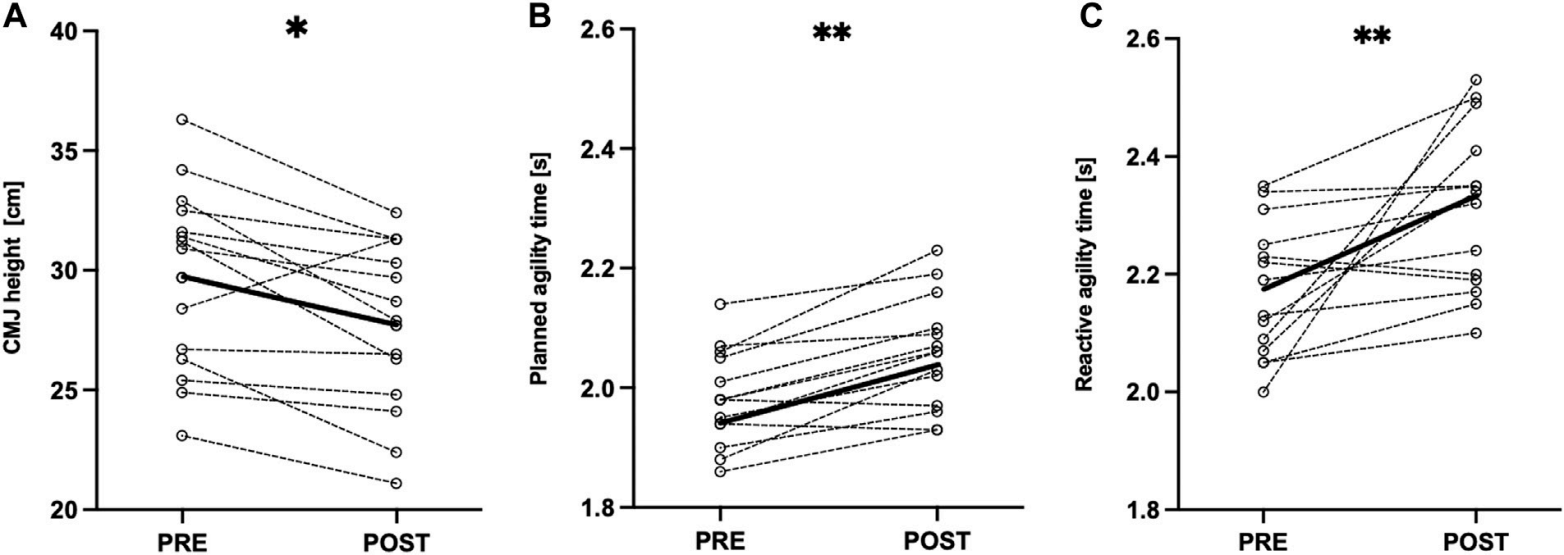
Individual pre- and post-SSG differences in **(A)** countermovement jump height; **(B)** planned agility time; **(C)** reactive agility time. Circles connected with dashed line indicate the individual data of each participant; black bold line indicates mean values. Significant changes between pre- and post-SSG assessments. *p < 0.05; **p < 0.01.

**FIGURE 4:**
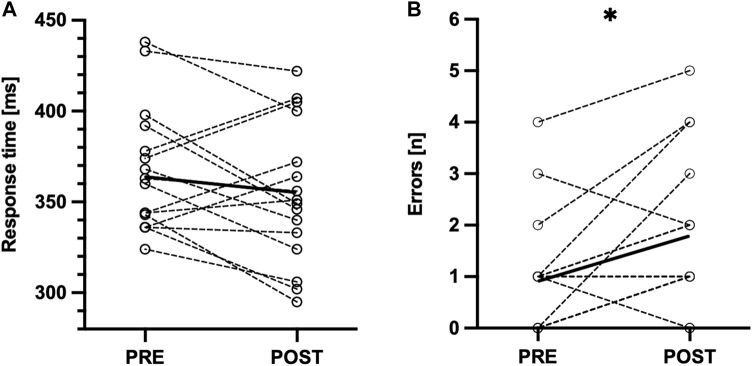
Individual pre- and post-SSG differences in Go/No-go task for **(A)** response time to “Go” stimuli; **(B)** incorrect responses to “No-go” stimuli. *p < 0.05.

After the SSG, a CMJ height decreased (−6.65% ± 6.23%, *p* = .014, g = .56, moderate effect, Table 3; Figure 3A), PA time (4.04% ± 2.53%, *p* = .002, most likely, g = .97, large effect, Table 3; Figure 3B), and RA time increased (6.45% ± 7.37%, *p* = .003, likely, g = 1.16, large effect, Table 3; Figure 3C). The response time in the GNG task did not change after the SSG (−3.36% ± 8.04%, *p* = .119, g = .29, small effect, Table 3; Figure 4A), whereas errors of commission increased (87.10% ± 138.99%, *p* = .023, r_c_ = .57, moderate effect, Table 3; Figure 4B).

**TABLE 3:** Pre- and post-SSG neuromuscular and perceptual-cognitive performance differences.

	PRE-SSG	POST-SSG	∆ MEAN	CI (90%)	SWC	∆ %	MBI	p	ES
VAS-F (A.U.)	21.31 ± 11.12	62.88 ± 9.87	41.56 ± 14.02	26.56, 43.44	2.80	41.56 ± 14.02	100/0/0	<.001	4.15^a^
							Most likely		
GNGt (ms)	366.73 ± 34.24	354.4 ± 38.9	−12.34 ± 28.63	−25.11, 0.73	5.89	−3.36 ± 8.04	1.4/18.7/79.9	.119	.29^a^
							Possibly		
GNGe (n)	0.93 ± 1.14	1.71 ± 1.48	0.81 ± 1.13	0.30, 1.32	0.28	87.10 ± 138.99	92.7/7.3/0.1	.023	.57^b^
							Likely		
CMJ (cm)	29.70 ± 3.65	27.72 ± 3.34	−1.98 ± 1.85	−2.85, −1.12	0.37	−6.65 ± 6.23	0.2/1.8/97.9	.014	.56^a^
							Likely		
PA (s)	1.98 ± 0.07	2.06 ± 0.09	0.08 ± 0.05	0.05, 0.10	0.02	4.04 ± 2.53	99.8/0.1/0	.002	.97^a^
							Most likely		
RA (s)	2.17 ± 0.11	2.31 ± 0.13	0.14 ± 0.16	0.06, 0.22	0.03	6.45 ± 7.37	99.2/0.8/0	.003	1.16^a^
							Most likely		

CI (90%), lower and upper confidence intervals; SWC, the smallest worthwhile change; ∆ %, the percentual difference between pre- and post-SSG, measurements; MBI, magnitude based interference; p, statistical significance; ES, effect size (a, Hedges’ g; b, Cohens’ r); VAS-F, visual analog scale of fatigue; GNGt, average Go/No-go task response time to “go” stimuli; GNGe, number of errors in Go/No-go task; CMJ, countermovement jump height; PA, planned agility time; RA, reactive agility time.

### 3.2 Relationship between changes in performance and exercise load

Significant correlations were found between ΔCMJ height and VHSR (r_s_ = .660, *p* = .006), ΔCMJ height and MSP (*r* = .536, *p* = .032, R^2^ = .286), ΔPA time and HACC (r_s_ = −.764, *p* = .002), ΔRA time and VHSR (r_s_ = −.501, *p* = .022), and ΔRA time and MSP (*r* = −.524, *p* = .037). In addition, ΔGNG errors correlated significantly with absolute and relative TDC (*r* = −.576, *p* = .021; *r* = −.631, *p* = .009; respectively), and ΔGNG response time with MSR (*r* = −.596, *p* = .015, R^2^ = .319). Moderate, large, and very large correlations between performance differences (Δ) and respective exercise load are shown in Table 4.

**TABLE 4:** Correlations and simple linear regressions between the exercise load variables and the performance differences (∆). Only moderate correlations are reported.

Dependent variable	Independent variable	r	p	CI (90%)	R^2^	SE
ΔCMJ	TDC (m)	.379^a^	.148	−.14, .74	.144	.028
	TDC (m/min)	.392^a^	.132	−.13, .74	.154	.836
	HSR	.384^a^	.142	−.14, .73	.147	.096
	VHSR	.660^b^	.006	.22, .88	—	—
	LMDEC	−.376^a^	.151	−.44, .53	.141	.951
	MSP	.536^a^	.032	−.05, .88	.286	.066
ΔGNGt	TDC (m)	−.323^a^	.223	−.71, .20	.104	.032
	TDC (m/min)	−.333^a^	.207	−71, 19	.111	.583
	LSR	.402^a^	.123	−.12, .75	.162	.038
	MSR	−.596^a^	.015	−.85, −.15	.319	.141
	LMACC	−.410^b^	.111	−.75, .10	—	—
	LMDEC	−.392^a^	.201	−.73, .16	.154	.016
	70%–79% HR_max_	−.345^a^	.191	−.18, .72	.119	.038
	>90% HR_max_	−.338^a^	.064	−.19, .71	.114	.041
ΔGNGe	TDC (m)	−.576^b^	.021	−.84, .10	—	—
	TDC (m/min)	−.631^b^	.009	−71, 19	—	—
	MSR	−.374^b^	.076	−.74, .17	—	—
	HSR	−.456^b^	.076	−.78, .07	—	—
	VHSR	−.370^b^	.159	−.74, .17	—	—
	HDEC	−.353^b^	.180	−.73, .19	—	—
	LMDEC	−.337^b^	.201	−72, .21	—	—
	MSP	−.531^b^	.053	−.82, .03	—	—
ΔPA	HACC	−.764^b^	.002	−.87, −.21	—	—
	LMDEC	.481^a^	.099	.09, .76	.230	.090
ΔRA	<59% HR_max_	.439^a^	.258	−.44, .55	.193	.078
	VHSR	−.501^b^	.022	−.32, .64	—	—
	MSP	−.524^a^	.037	−.81, −.04	.275	.214

ΔCMJ, countermovement jump height; ΔPA, planned agility time; ΔRA, reactive agility time; ΔGNGt, average Go/No-go task response time to “go” stimuli; Δ GNGe, number of errors in Go/No-go task; TDC (m), total distance covered; TDC (m/min), relative total distance covered; HR_max_ (%), time spent in respective heart rate zone; LSR, low-speed running (0–6.99 km/h); MSR, medium-speed running (7–13.99 km/h); HSR, high-speed running (14–20.99 km/h); VHSR, very high-speed running (>21 km/h); LMACC, low to moderate accelerations (1–2.99 m/s); LMDEC, low to moderate decelerations (1–2.99 m/s^2^); HACC, high-intensity accelerations (>3 m/s); HDEC, high-intensity decelerations (>3 m/s); MSP, maximal speed achieved during SSG (km/h); r, correlation coefficient (a, Pearson’s correlation coefficient; b, Spearman’s correlation coefficient); p, level of significance; CI (90%), lower and upper confidence intervals; R^2^, the amount of variance explained; SE, standard error of regression coefficient.

## 4 Discussion

As shown, exercise load in SSG leads to a significant increase in youth players’ subjective perception of fatigue. Considering fatigue as a multifactorial process, it may interfere with physical, mental, metabolic, morphological, and biochemical alterations, among others (Mohr et al., 2005). The exercise load included in the SSG led to changes in the neuromuscular and perceptual-cognitive performance of youth soccer players. More specifically, their explosive strength, planned and reactive agility, and accuracy in the response inhibition task were significantly affected by the 30-min SSG 4v4 protocol. These deteriorations were not significantly associated with neither internal nor external load variables. However, a less pronounced drop in agility performance and explosive strength after SSG was related to some variables of external load such as HACC, VHSR, and MSP. This indicates that the high-intensity efforts performed during the SSG could, to some extent, compensate for their decrements in youth soccer players. The deterioration of reactive agility and accuracy in response inhibition task points to the fact that SSG affects not only neuromuscular but also sensory and cognitive components of players’ motor performance.

### 4.1 Effects of SSG on neuromuscular performance

Neuromuscular performance was affected by SSG in terms of decreased CMJ height and increased PA time. These reductions can be ascribed to players’ high mechanical load in SSGs produced by numerous changes in speed and direction. This requires the eccentric strength of knee extensors to reduce movement velocity in the deceleration phase, and concentric strength in the acceleration phase (Jones et al., 2009). Repeated muscle contractions produce muscle damage which may temporarily decrease players’ force production (Greig and Siegler, 2009; Thorlund et al., 2009). In addition, the fast transition from concentric to the eccentric phase of movement relies on a muscle and tendon complex utilizing a stretch-shortening cycle (Nicol et al., 2006). Fatigue induced by mechanical load alters the neuromuscular activation patterns of human skeletal muscle (Gollhofer et al., 1987). Thus, the observed declines in CMJ height (−6.65%) are in agreement with Rebelo et al. (2016) who reported decreased CMJ height (−9.4%) after two sets of three repetitions of 6-min SSG 4v4. Bujalance-Moreno et al. (2020) applied four 4-min game intervals separated by 2-min passive recoveries between the bouts. Significant changes were observed in the linear 20-m sprint time (1.3%) but not in the 5-m sprint and CMJ height. A different result could be explained by a lower overall work time in the SSG protocol compared to ours.

Furthermore, repeated high-intensity efforts tend to decrease the power generated by hamstring and gluteal muscle groups (Small et al., 2010; Edouard et al., 2018) and affect the change of direction speed by impaired lower-limbs biomechanics (Cortes et al., 2012). These presumptions point to the increments of PA and RA times, which were observed in our study (4.04% and 6.45%; respectively). It is suggested that neuromuscular performance decrease is likely associated with the number of accelerations and decelerations. However, we did not find significant relationships between the number of high-intensity accelerations and decelerations with neither CMJ height nor PA time deterioration. On the other hand, moderate correlations were observed in low to moderate intensity decelerations with ΔCMJ height (*r* = −.376) and ΔPA time (*r* = .481). This may be ascribed to 4v4 SSGs’ pitch dimensions which do not allow players to cover enough high-intensity distances and perform high-intensity accelerations and decelerations.

It needs to be stated that SSGs are adjustable in load variables, e.g., number of players, pitch size, rules, or work/rest duration (Sarmento et al., 2018). These factors influence the internal and external load of players (Hill-Haas et al., 2011), resulting in their differential response. Specifically, one study suggested that small formats of SSG (i.e., 1v1 and 3v3) do not have a significant impact on the lower limb power in CMJ. Even though these players surpassed our subjects in the relative distance covered in the game (m/min^−1^) and the internal load (%HR_max_), probably a short exercise duration could not produce a sufficient external load to induce neuromuscular fatigue (Clemente et al., 2017). A lower number of repetitions and longer resting periods compensate for players’ neuromuscular performance decrement. This can also be achieved by reducing the pitch size of SSGs (Castillo et al., 2019). The question remains whether declines in reactive agility and perceptual-cognitive performance can be compensated as well.

### 4.2 Effects of SSG on perceptual-cognitive performance

A slight decline in players’ response time (−3.36%, n.s.) but a significant increase in errors of commission (87.1%) was observed in the response inhibition task. SSGs engage players’ physical and mental effort in an open-skill and dynamic environment (Owen et al., 2012; Mitrotasios et al., 2021). Therefore, some aspects of cognition are affected by sport-specific exercises as well. Previously, an improved inhibitory control was found in primary school children after the high-intensity SSG (Lind et al., 2019). Exercise-induced arousal improves a choice reaction time (Kashihara and Nakahara, 2005), but it can simultaneously affect object detection in sport-specific tasks (Klatt and Smeeton, 2021). These findings support our results, as youth soccer players react to visual stimuli slightly faster but less accurately after the SSG.

Intermittent exercise induces changes in perceptual-cognitive processes in both high- and low-level soccer players (Casanova et al., 2013). These temporal cognitive declines are often ascribed to the hypothesis of hypofrontality. For the maintenance of motor functions, the brain is limiting its resources to movement centers, causing less activation in centers responsible for higher-order cognitive functions (Dietrich, 2006). However, the lack of advanced neuroimaging tools available for use in sport-specific conditions does not allow us to reveal these changes. More evident is the role of mental fatigue and its impairment of decision-making, the tactical aspect of performance, and the skill execution in elite athletes (Russell et al., 2019). It has been shown that even a 20-min intermittent soccer-specific exercise produces mental fatigue in well-trained soccer players (Bian et al., 2022). However, research needs to shed light on the inducement of mental fatigue by SSGs.

Cognition also plays an important role in reactive agility performance (Young et al., 2015). It is suggested that reactive and planned agility differentiate under fatigue conditions (Ciocca et al., 2022). This may be corroborated by our results with the largest effect size for ΔRA time (6.45%, g = 1.16). Both the Y-shaped reactive agility test and the go/no-go task partly focus on the assessment of decision-making ability. This ability is often evaluated in soccer by the analysis of in-game successful passing (i.e., decision-making index) which tends to decrease with an increasing volume of exercise (Mitrotasios et al., 2021) and additional inducement of mental fatigue before SSG (Fortes et al., 2019; Gantois et al., 2020). Our results confirm that changes in perceptual-cognitive performance occur in response to sport-specific exercise load. However, the current literature deals with the lack of information about the effects of exercise on reactive agility while preferring more conventional methods of planned agility assessments (Marqués-Jiménez et al., 2022; Bilić et al., 2023).

### 4.3 Relationship between load variables and performance changes

No single robust variable of SSG load was related to neuromuscular nor perceptual-cognitive performance declines in youth soccer players. In the case of external load, only a ΔPA time was moderately correlated with the amount of LMDEC. This resulted in a 23% proportion of variance in ΔPA time increase. It can be assumed that players in our study were not able to cover enough high-intensity distance and perform high-intensity accelerations or decelerations, which would point to these variables as the most contributing to respective performance declines. From the perspective of internal load, time spent in <59% HR_max_ moderately correlated with ΔRA time by a 19% proportion of variance. The discovered relationships did not fully reveal the contribution of exercise load to the observed declines in neuromuscular and perceptual-cognitive performance.

Previously, a significant association was found between CMJ height decline and high-intensity activities in SSG 4v4 (Rebelo et al., 2016). Similar associations were presented in relation to the amount of external load in soccer matches (Rampinini et al., 2011; Silva et al., 2013; Rowell et al., 2017). Since SSGs 4v4 can relatively overload the mechanical work accumulated in a soccer match (Lacome et al., 2018), similar associations were expected to be found in our case. As mentioned above, we did not confirm these presumptions. Soccer games also include barely detectable high-power actions without a change in the location of players, such as jumping or duels (Dalen et al., 2016). However, our findings can be related to the fact that amateur and professional players’ kinematic profiles in SSGs differ in the amount of high-intensity actions (Dellal et al., 2011). Additionally, no correlation was reported between changes in linear sprint performances and the rate of perceived exertion registered during the four different 5v5 SSG protocols (Castillo et al., 2019). It is also suggested that interindividual differences exist between the perceived effort and the lactate responses of players during SSGs (Köklü et al., 2015). In accordance with our results, the evaluation of youth players’ level of fatigue or the response to exercise should incorporate more than subjective assessment methods.

From the perspective of external load, players who achieved a higher VHSR distance and MSP in SSG tend to show a lesser drop in the ∆CMJ height. Similarly, a correlation of HACC with ∆PA time, and ∆RA time with VHSR was found. A slight compensatory effect occurred in players who were able to achieve very high-speed running and high-intensity accelerations. The same was true for the total distance covered in SSG and the errors in the response inhibition task. In fact, the usage of SSG, usually in warp-ups, can also have positive effects on CMJ and reactive agility performance (Zois et al., 2011). Temporally enhanced performance can be explained by athletes’ physiological response to physical exercise in terms of increased muscle temperature and blood flow, increased neural activation, and improved force-velocity relationship (Binkhorst et al., 1977; Sale, 2002). The lack of correlation in the case of perceptual-cognitive and reactive agility performance with load variables would be attributed to the complexity of SSGs as they engage humans’ motor, sensory, and cognitive systems (Owen et al., 2012; Figueira et al., 2019). Therefore, it seems unlikely to reveal a single variable that would interfere with cognitive performance declines solely from load tracking data or subjective assessment of players.

Nevertheless, this study has some limitations. Heterogeneity in study protocols, the performance level or age of participants makes it difficult to compare our results with the findings of other authors. In addition, there is a variance in the assessment procedures regarding to agility and perceptual-cognitive performance testing. Larger sample sizes and consideration of the growth and maturity levels of players should bring a more profound understanding of the acute effects of SSGs on reactive agility and perceptual-cognitive performance. The novel objective cognitive function evaluation methods could provide evidence to support temporal cognitive changes in response to acute bouts of sport-specific exercises. Since the mental effort of sport-specific tasks is often neglected, research would develop a method that counts physical and psychological exertion of players in these tasks. Practitioners could better estimate the load of sport-specific exercises, players’ level of fatigue, and thereby optimize the exercise load in training microcycles.

## 5 Conclusion

Fatigue induced by SSG has the most negative effect on reactive agility, followed by planned agility, and explosive strength in youth soccer players. It also affects decision-making in response inhibition task rather than speed of response to visual stimuli. There are no significant relationships between the external load variables and neuromuscular performance declines. The accuracy of decision-making is not affected by the internal load. Interestingly, high-intensity actions performed during SSG (i.e., very high-speed running, high-intensity accelerations) partially compensate for fatigue-induced declines in agility and explosive strength. Since load variables and the visual analog scale of fatigue were not able to fully reveal changes in players’ performance, further research should aim to develop a method for assessing both the physical and cognitive components of exercise load in sport-specific tasks, which would also reflect players’ level of fatigue. Consequently, besides the acute neuromuscular performance declines, less accurate decision-making and slower change of direction speed in response to visual stimuli can be expected after the application of SSG 4v4 in training.

### 5.1 Practical applications

We suggest using the SSG 4v4 and its numerous modifications to target players’ agility and decision-making ability. However, practitioners should be aware of subsequent acute performance declines in the planning of the training structure. These declines are hardly detectable by the available load tracking systems and subjective methods. The application of SSGs with a relatively high work-to-rest ratio must be manipulated with caution for youth soccer players.

## Data Availability

The original contributions presented in the study are included in the article/supplementary material, further inquiries can be directed to the corresponding author.
